# A Retrospective Study of Acute Renal Failure in Children: Its Incidence, Etiology, Complications and Prognosis

**DOI:** 10.7759/cureus.1274

**Published:** 2017-05-25

**Authors:** Kadar Ismail Hassan, Jama Hodan M, Chunfu Li

**Affiliations:** 1 Department of Paediatrics, Nanfang Hospital, Southern Medical University, Guangzhou, P.R.China; 2 Department of Nephrology, Hargeysa Group Hospital, Hargeysa City, Somalia.

**Keywords:** acute renal failure, incidence, etiology, clinical presentation, complication and outcome

## Abstract

**Background:**

Acute renal failure (ARF) developed due to various causes and may lead to significant morbidity and mortality among pediatric patients.

**Objectives:**

The study was conducted to determine the incidence, etiology, outcome of treatment and clinical presentation of ARF in pediatric patients in Somalia.

**Methods:**

Comprehensive case history of 39 pediatric patients below 12 years of age, admitted with renal diseases in four tertiary care hospitals in Hargeisa and Borama cities in Somalia during December 2015 to November 2016. They were subjected to clinical investigation and laboratory test analysis based on the inclusion criteria of renal insufficiency characterized by serum creatinine level more than 1.5 mg/dl.

**Results:**

ARF was most commonly found in five to 12 years age group (53.8%) compared to infant (zero to one year) and pre-school (one to five years) children (23.08%). Mean age of presentation was 6.14 years. Male female ratio in this study was 1.2: 1. Most common presenting clinical feature in our study was oliguria (97.43%), swelling (69.2%), fever (84.1%), abdomen pain and nausea-vomiting (41.02%). Common clinical signs were edema (66.66%), altered sensorium (51.28%), hematuria (48.71%) and hypertension (38.46%). Snake bite and acute post streptococcal glomerulonephritis were the two most common causes of ARF in children in our study. Common complications were hypertension (38.46%), anemia (35.89%), hyperkalemia (25.64%) and infection (20.51%), all of which were within the previously reported range. The factors which correlated positively with increased mortality and morbidity were females with age below one year , etiology like septicemia and systemic lupus erythematosus (SLE), high peak serum creatinine concentration and complicated by disseminated intravascular coagulation (DIC).

**Conclusion:**

Many causes of ARF are preventable and it should be possible to reduce mortality and morbidity due to ARF through purposive preventive measure and availability of the better medical facility.

## Introduction

Acute renal problems constitute a large proportion of hospital admission and outpatient attendance among pediatric population. Acute renal failure (ARF) in children, compared with adults, are often reported with speedy recovery without any residual effect and complications. Among the hospitalized patients, ARF may often lead to life-threatening conditions and it is regarded as one of the major cause of significant morbidity and mortality among pediatric patients. Five to 10% of patients in pediatric intensive care unit (PICU) have evidence of renal insult and ARF occurs in 8% of neonates in the neonatal intensive care unit (NICU) [1-2].

Despite the major advance in the PICU tools and renal replacement therapy (RRT) methods, the reported mortality rate among inpatients ARF is 29% to 46% [3-4]. The high frequency of occurrence and substantial morbidity and mortality of ARF demands a logical approach to its prevention and early diagnosis and the prompt recognition and management of its complication. Therefore, the emphasis on prevention remains cardinal but early detection and appropriate treatment also could provide complete recovery which is a major goal of ARF therapy.

The ARF is an abrupt cessation of kidney function with life-threatening consequences [5] in association with significant multiple organ system failure (MOSF) [6], the absence of consensus definition of ARF led to significant confusion both in clinical practice and in the medical literature. In 2004, the Acute Dialysis Quality Initiative (ADQI) group published the RIFLE (Risk, Injury, Failure, Loss of kidney function and End-stage kidney disease) classification of ARF that helped in early detection of renal insult before progression to ARF and thus reduced the morbidity and mortality. Moreover, the term acute kidney injury (AKI) was preferred rather than ARF and the criteria for AKI definition was formulated by the Acute Kidney Injury Network (AKIN) [7].

According to AKIN, the following criteria of an abrupt (within 48 hours) reduction in kidney functions are characterized by i) absolute increase in serum creatinine more than or equal to 0.3 mg/dl from baseline or ii) increase in serum creatinine more than or equal to 50% (1.5 fold from baseline) or iii) reduction in urine output (documented oliguria of less than 0.5 ml/kg / hour for more than six hours). But, in most of the cases, the baseline creatinine is not available. ARF may be non-oliguric or even polyuric in 10% to 15% of cases, which may lead to misdiagnosis on clinical assessment if we rely on daily urine volume only [8]. Due to the advancement in complex treatment strategy, children treated with acute and chronic illness associated with ARF may develop inexplicable complications. Hence, it is essential and critical to re-evaluate the incidence, etiology, presentation, complication and outcome in pediatric ARF which may further aid in revising the preventive strategies and implementing appropriate supportive care. Informed consent statement was obtained for this study.

## Materials and methods

### Study area and population

The children aged 12 years or less, suffering from ARF symptoms were enrolled between December 2015 and November 2016 in pediatric medicine department of four tertiary care hospitals in Hargeisa and Borama cities in Somalia: Hargeisa Group Hospital and Garger Hospital in Hargeisa and Al-Hayat Hospital in Borama, and Emirates Hospital in Hargeisa. The subjects were included in this study based on the inclusion criteria of renal insufficiency characterized by serum creatinine level more than 1.5 mg/dl and were excluded showing the history of any previously reported renal disease. Distinct parameters were characterized such as parameters of incidence, etiology, presentation, complications and clinical outcome. The data collected from these cases were then analyzed. 

### Clinical and laboratory investigations

All ARF patients in this study were examined clinically along with proper and complete history recording and subjected to laboratory investigations like serum levels of urea, creatinine, sodium, potassium, cholesterol, triglycerides and with complete blood count, routine and microscopic urinary examination. Other special investigations like ultrasonography (USG), antinuclear antibody (ANA), anti-double-stranded deoxyribonucleic acid (anti- dsDNA), anti-streptolysin O titer (ASO), complement component 3 (C3) level and kidney biopsy were performed whenever necessary. 

### Study design

A prospective observational study was undertaken to evaluate the percentage of incidence of ARF among children hospitalized for all causes, the differential etiology of ARF, commonly presented clinical features, age, and sex ratio among children with ARF, and also to delineate the clinical complications and prognosis of treatment and management of cases.

## Results

###  Incidence ratio and etiological distribution of ARF in children

**Figure 1:**
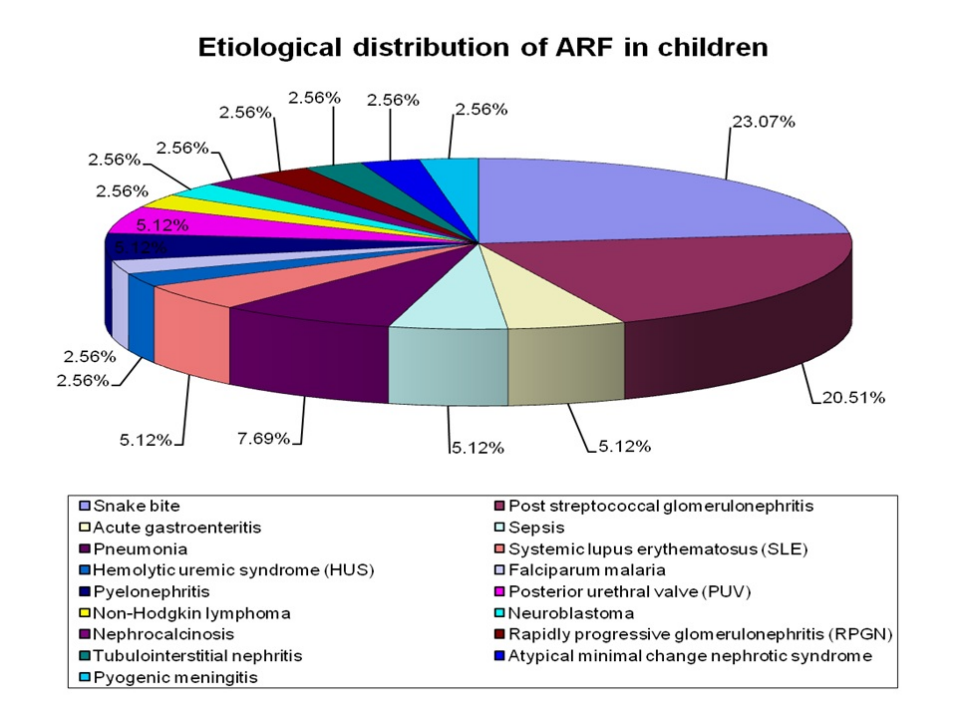
Etiological distribution of acute renal failure among children

In the studied period, a total of 1520 children were admitted in pediatric medicine ward for the different illness and 39 cases were confirmed as ARF. Therefore, the incidence of ARF among hospitalized children in our hospitals was 2.6%. The most common cause of ARF in children in our study was snakebite (nine cases, 23%,) followed by post-streptococcal glomerulonephritis (eight cases, 20.5%). Three cases were due to pneumonia and two cases each were due to gastroenteritis, sepsis, systemic lupus erythematosus (SLE), pyelonephritis and posterior urethral valve (PUV). Other cases were due to falciparum malaria, nephrocalcinosis, rapidly progressive glomerulonephritis, non-Hodgkin lymphoma, neuroblastoma, hemolytic uremic syndrome, pyogenic meningitis, atypical minimal change nephrotic syndrome and tubulointerstitial nephritis (Table 1-Figure 1). 

**Table 1:** Etiological distribution of acute renal failure (ARF) among children

No.	Etiology of ARF	No. of cases	Percentage of total cases
1	Snake Bite	9	23.07%
2	Post streptococcal glomerulonephritis	8	20.51%
3	Acute gastroenteritis	2	5.12%
4	Sepsis	2	5.12%
5	Pneumonia	3	7.69%
6	Systemic lupus erythematosus (SLE)	2	5.12%
7	Hemolytic uremic syndrome (HUS)	1	2.56%
8	Falciparum malaria	1	2.56%
9	Pyelonephritis	2	5.12%
10	Posterior urethral valve (PUV)	2	5.12%
11	Non-Hodgkin lymphoma	1	2.56%
12	Neuroblastoma	1	2.56%
13	Nephrocalcinosis	1	2.56%
14	Rapidly progressive glomerulonephritis (RPGN)	1	2.56%
15	Tubulointerstitial nephritis	1	2.56%
16	Atypical minimal change nephrotic syndrome	1	2.56%
17	Pyogenic meningitis	1	2.56%

### Age and sex distribution of different etiological factors

**Figure 2:**
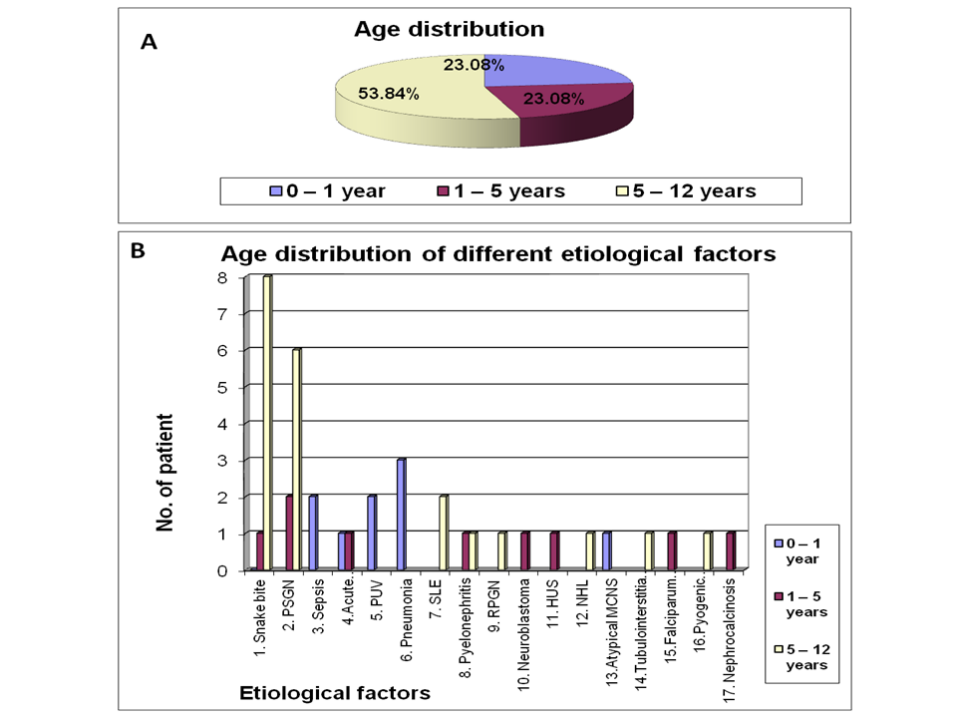
Age distribution of different etiological factors in acute renal failure patients

According to the age distribution of cases, nine cases (23.08%) were admitted in zero to one year age group (age up to one year included). Similarly, nine cases (23.08%) were admitted in one to five years age group and 21 cases (53.84%) were admitted to five to 12 years age group (Table 2-Figure 2A-2B). 

**Table 2:** Age distribution of different etiological factors

Etiology	0 – 1 year	1 – 5 years	5 -12 years	Total
1. Snake bite	0	1	8	9
2. PSGN		2	6	8
3. Sepsis	2			2
4. Acute gastroenteritis	1	1		2
5. PUV	2			2
6. Pneumonia	3			3
7. SLE			2	2
8. Pyelonephritis		1	1	2
9. RPGN			1	1
10. Neuroblastoma		1		1
11. HUS		1		1
12. NHL			1	1
13. AtypicA typically change nephrotic syndrome (MCNS)	1			1
14. Tubulointerstitial nephritis			1	1
15. Falciparum malaria		1		1
16. Pyogenic meningitis			1	1
17. Nephrocalcinosis		1		1
Total	9 (23.08%)	9 (23.08%)	21 (53.84%)	

**Figure 3:**
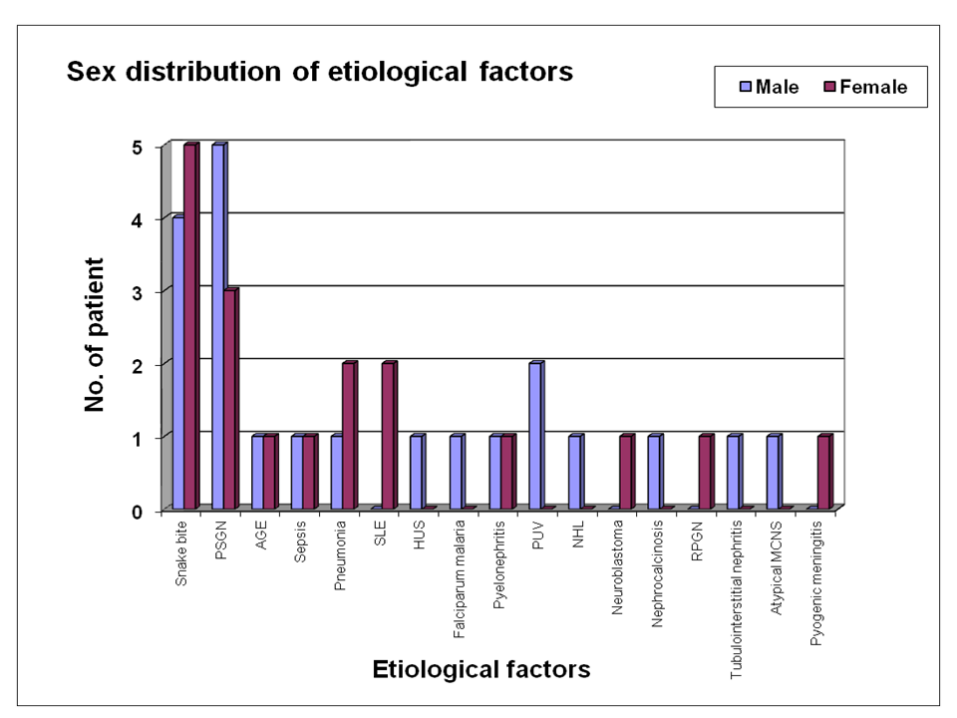
Sex distribution of different etiological factors in acute renal failure patients

Twenty-one cases (53.85%) were male and 18 cases (46.15%) were female children in our study with a male to female ratio of 1.2: 1. Most common cause of the ARF in the male patient was post-streptococcal glomerulonephritis (five case, 23.8%) followed by snakebite (four case, 19.04%), posterior urethral valve (two case, 9.52%) and one case each were due to ten others different etiologies (Table 3-Figure 3). 

In female patients, most common cause was snakebite (five cases, 27.77%) followed by acute post-streptococcal glomerulonephritis (three cases, 16.66%), systemic lupus erythematosus (two cases, 11.11%), pneumonia (two cases, 11.11%) and one case each were due to six other etiologies (Table 3). No case was due to hemolytic uremic syndrome, falciparum malaria, nephrocalcinosis, non-Hodgkin lymphoma (NHL), atypical minimal change nephrotic syndrome, tubulointerstitial disease, posterior urethral valve etc. Male and female patient in different etiological factor is depicted in Figure 3.

**Table 3:** Sex distribution of etiological factors

No.	Etiology of ARF	No. of cases	Male	Female
1	Snake bite	9	4	5
2	Poststreptococcal glomerulonephritis (PSGN)	8	5	3
3	Acute gastroenteritis (AGE)	2	1	1
4	Sepsis	2	1	1
5	Pneumonia	3	1	2
6	Systemic lupus erythematosus (SLE)	2	0	2
7	Hemolytic uremic syndrome (HUS)	1	1	0
8	Falciparum malaria	1	1	0
9	Pyelonephritis	2	1	1
10	Posterior urethral valve (PUV)	2	2	0
11	Non-Hodgkin lymphoma (NHL)	1	1	0
12	Neuroblastoma	1	0	1
13	Nephrocalcinosis	1	1	0
14	Rapidly progressive glomerulonephritis (RPGN)	1	0	1
15	Tubulo interstitial nephritis (TIN)	1	1	0
16	Atypical minimal change nephrotic syndrome	1	1	0
17	Pyogenic meningitis	1	0	1

The mean age of male patients was 6.12 years and for the female patient was 6.15 years. Therefore, in male and female cases, mean age was almost the same. The mean age of each etiological group is given in Table 4. Mean age in snakebite patients was 8.88 years, in post-streptococcal glomerulonephritis (PSGN) cases was 8.47 years and in systemic lupus erythematosus (SLE) was 12 years. So, all the three conditions were more common in older children. On the other hand, mean age in sepsis was 5.5 month and in pneumonia was 3.5 months which were reported as the most common clinical conditions in small infants. 

**Table 4:** Mean age in different etiological groups

No.	Etiology of ARF	Mean age
1	Snake bite	8.88 years
2	Post streptococcal glomerulonephritis (PSGN)	8.47 years
3	Acute gastroenteritis (AGE)	1.8 years
4	Sepsis	5.5 months
5	Pneumonia	3.5 months
6	Systemic lupus erythematosus (SLE)	12 years
7	Hemolytic uremic syndrome (HUS)	3 years
8	Falciparum malaria	4.5 years
9	Pyelonephritis	6 years
10	Posterior urethral valve (PUV)	23.5 days
11	Non-Hodgkin lymphoma (NHL)	7 years
12	Neuroblastoma	3 years
13	Nephrocalcinosis	2.5 years
14	Rapidly progressive glomerulonephritis (RPGN)	7 years
15	Tubulointerstitial nephritis (TIN)	10 years
16	Atypical minimal change nephrotic syndrome	1 year
17	Pyogenic meningitis	10 years

### Clinical presentation of the patients

**Figure 4:**
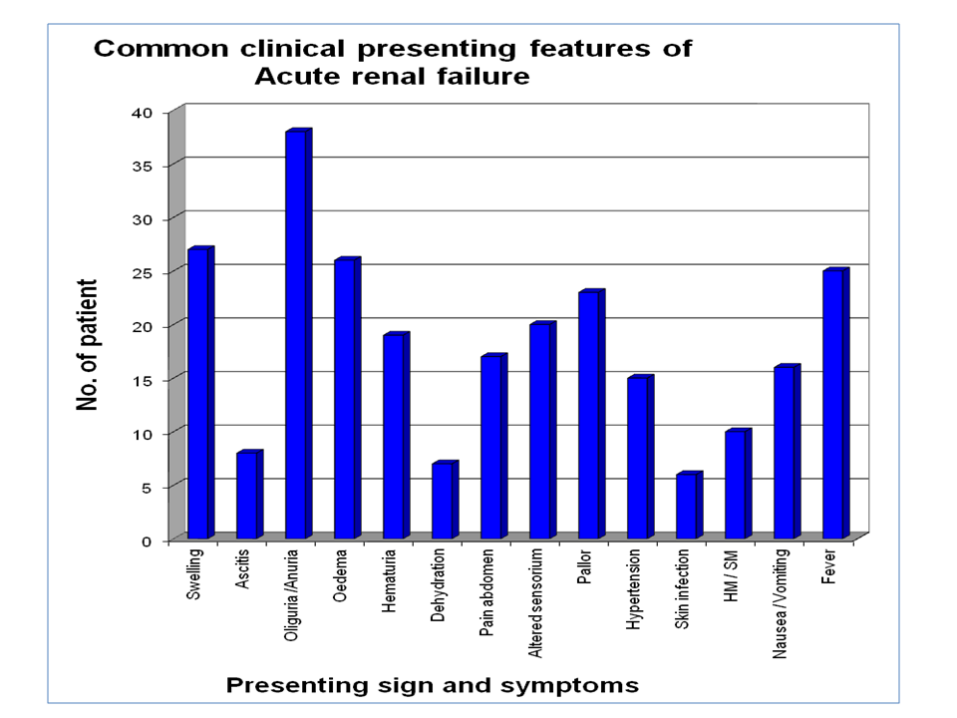
Common clinical presenting features of acute renal failure in patients

In this study, the most common presenting symptom was oliguria (38 cases, 97.43%). Oliguria was present in all cases except one, which was the non-oliguric renal failure due to tubulointerstitial nephritis. Other common presenting symptoms were swelling (27 cases, 69.23%), fever (25 cases, 64.10%), abdomen pain (17 cases, 43.58%), nausea or vomiting (16 cases, 41.02%). Common presenting clinical signs were edema (26 cases, 66.66%), altered sensorium (20 cases, 51.28%), hematuria (19 cases, 4.71%), hypertension (15 cases, 38. 46%), hepato-splenomegaly (10 cases, 25.64%) and others. Different presentation of ARF is given in Table 5 and graphically depicted in Figure 4. 

**Table 5:** Presentation of acute renal failure (ARF)

No.	Etiology	Swelling	Ascitis	Oliguria /Anuria	Oedema	Hematuria	Dehydration	Pain abdomen	Altered sensorium	Pallor	Hypertension	Skin infection	Hepatomegaly (HM) / Splenomegaly (SM)	Nausea / Vomiting	Fever
1	Sepsis	-	-	2	-	-	2		2	2	-				
2	PSGN	8	3	8	8	7		1	1	3	7	4	3	4	2
3	Acute gastroenteritis			2			2	2	2	-	-	-	-	1	4
4	PUV			2	-	1	-	-	2	-	-	-		-	2
5	SLE	1	1	2	1	8		2		2		1	2	1	1
6	Snake bite	9		9	9	-		5	7	6	1	1	1	3	1
7	RPGN	1	1	1	1	-	-	-	-	1	1	-	1	-	2
8	Tubulointerstitial nephritis	1		-	1	-	-	-	-	1	-	-	-	1	1
9	Atypical MCNS	1	1	1	1	-	-	1	-	-	1	-	-	-	1
10	Pyogenic meningitis	-	-	1	-	-	1	-	1	-	-	-	-	1	1
11	Pneumonia	-	-	3	-	-	2	-	1	2	-	-	-	1	3
12	HUS	1	-	1	1	1	-	1	1	1	-	-	1	1	1
13	NHL	1	-	1	1	-	-	1	1	1	1	-	-	1	1
14	Nephroca-lcinosis	1	1	1	1	-	-	1	1	1	-	-	-	-	1
15	Neuroblastoma	1	-	1	1	-	-	1	-	1	1	-	-	-	1
16	Falciparum malaria	-	-	1	-	1	-	-	1	1	1	-	1	-	1
17	Pyelonephritis	2	1	2	1	1	-	2	-	1	2		1	2	2
Total		27	8	38	26	19	7	17	20	23	15	6	10	16	25

**Figure 5:**
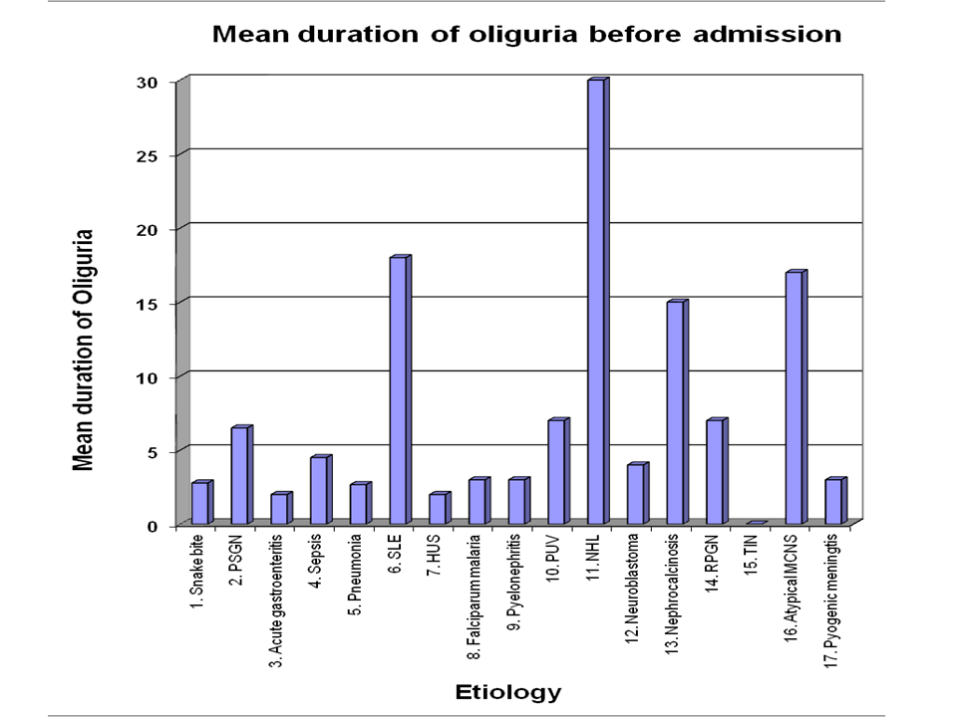
Mean duration of oliguria before admission in acute renal failure patients

Mean duration of oliguria was less than 10 days in most etiological cases. Mean oliguria for more than 10 days was present in three etiological groups. A typical minimal change nephrotic patients had oliguria for 17 days. Mean oliguria of two SLE patients was 18 days. The non-Hodgkin lymphoma (NHL) child had oliguria for one month (Table 6-Figure 5). 

**Table 6:** Duration of oliguria before admission

Etiology	Mean Duration of Oliguria
1. Snake bite	2.78 days
2. PSGN	6.5 days
3. Acute gastroenteritis	2 days
4. Sepsis	4.5 days
5. Pneumonia	2.67 days
6. SLE	18 days
7. HUS	2 days
8. Falciparum malaria	3 days
9. Pyelonephritis	3 days
10. PUV	7 days
11. NHL	30 days
12. Neuroblastoma	4 days
13. Nephrocalcinosis	15 days
14. RPGN	7 days
15. Tubulointerstitial nephritis (TIN)	No oliguria
16. Atypical MCNS	17 days
17. Pyogenic meningitis	3 days

### Complication in the course of acute ARF in patients

**Figure 6:**
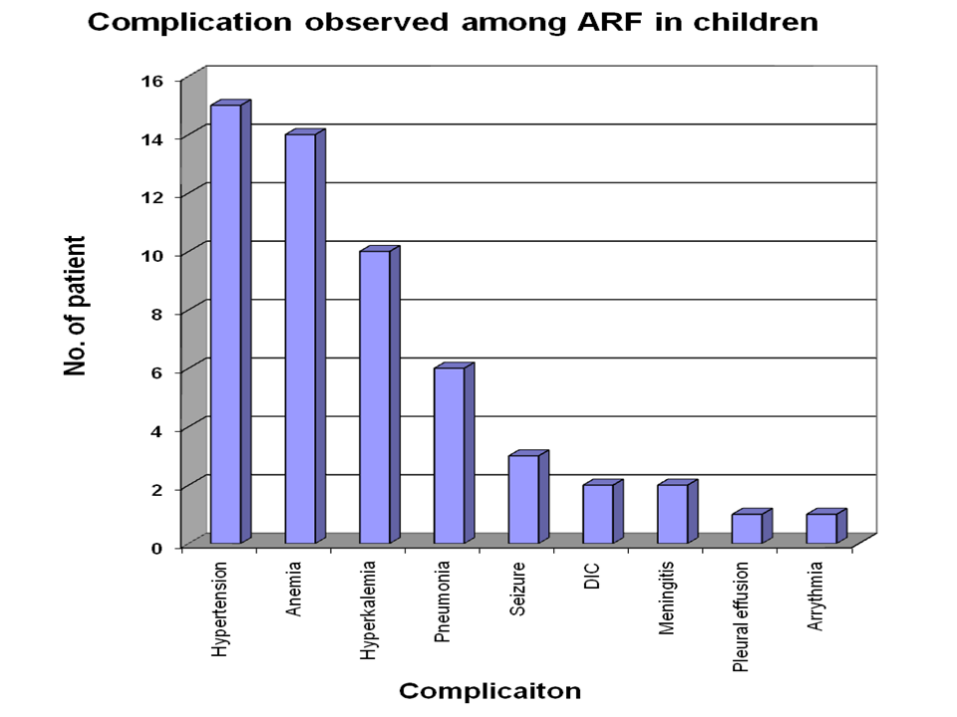
Complications in acute renal failure patients

The most common complications among all during the course of acute illness from ARF in the children was hypertension (15 cases, 53.6%). Other complications reported were anemia (14 cases, 50%), hyperkalemia (10 cases, 35.71%), pneumonia (six cases, 21.42%), seizure (three cases, 10.71%), disseminated intravascular coagulation (DIC) (7.14%), meningitis (two cases, 7.14%), arrhythmia (one case, 3.57%) and pleural effusion (3.57%). Eleven cases were reported without any complications (Figure 6). 

### Outcome of clinical complications in patients

**Figure 7:**
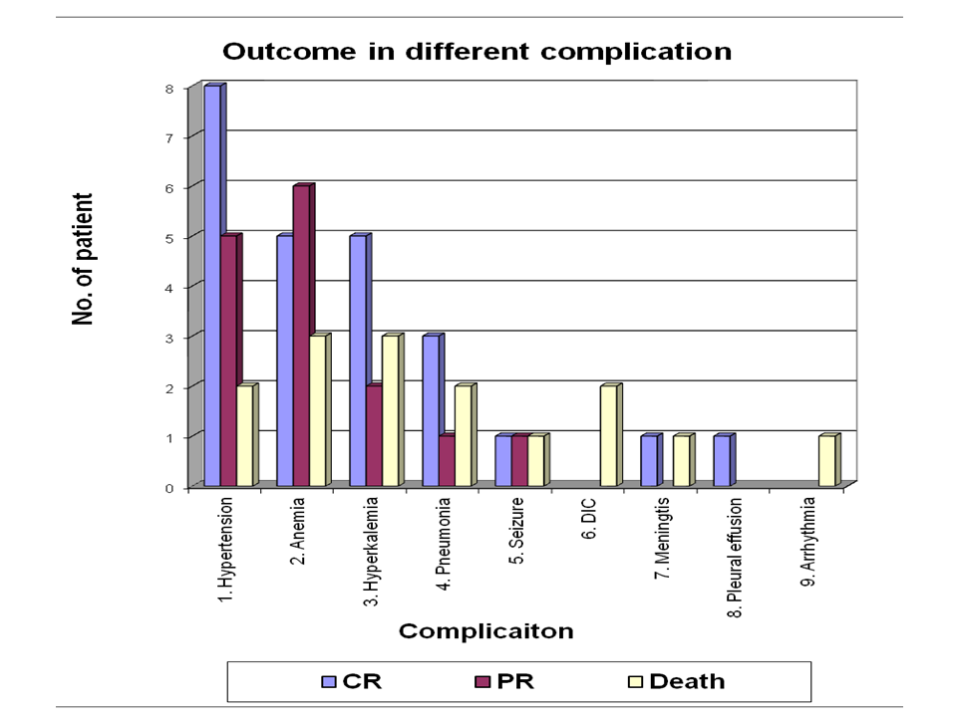
Outcome of different complications in acute renal failure patients

Among the children, who developed hypertension as a complication, 53.3% recovered completely, 33.3% recovered partially and 13.3% died. Anemia was common in the partially recovered group (42.85%). Among the 14 cases that developed anemia as a complication, five patients (35.7%) completely recovered and three cases (21.4%) died. Hyperkalemia developed in 10 cases of ARF. Fifty percent of hyperkalemia patient completely recovered, three patients (30%) died and two patients (20%) partially recovered. Two patients developed disseminated intravascular coagulation (DIC) and both of them died. Of the various infective complications, six patients (75%) developed pneumonia and two patients developed meningitis (25%). Other complications in ARF patient and their outcome are given in Table 7-Figure 7. 

**Table 7:** Outcome in different complications CR- complete recovery. PR- partial recovery.

Complication	CR	PR	Death	Total
1. Hypertension	8 (53.3%)	5 (33.3%)	2 (13.3%)	15
2. Anemia	5 (35.7%)	6 (42.85%)	3 (21.4%)	14
3. Hyparkalemia	5 (50%)	2 (20%)	3 (30%)	10
4. Pneumonia	3 (50%)	1 (16.6%)	2 (33.3%)	6
5. Seizure	1 (33.3%)	1 (33.3%)	1 (33.3%)	3
6. DIC	-	-	2 (100%)	2
7. Meningitis	1 (50%)	-	1 (50%)	2
8. Pleural effusion	1 (100%)	-	-	1
9. Arrhythmia	-	-	1 (100%)	1

### Prognosis of treatment and follow-up of patients

**Figure 8:**
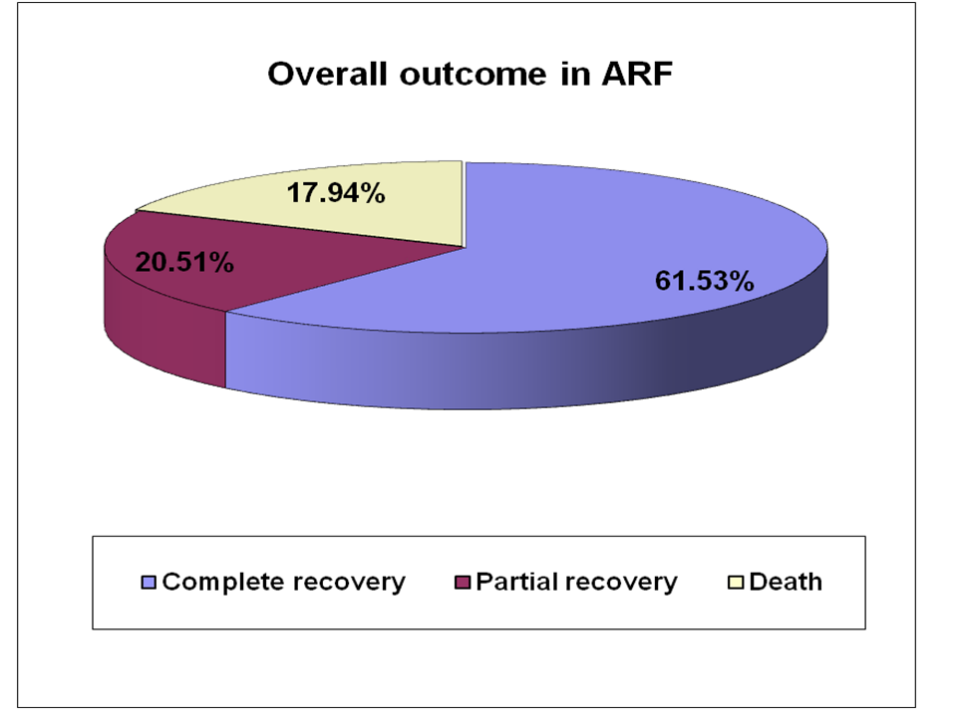
Overall outcome in acute renal failure cases

A total 39 cases were included and follow up has been done for a short period of two weeks. Among them, 24 cases (61.53%) recovered from ARF completely, and eight cases (20.51%) recovered partially with some persistent biochemical abnormality during discharge and follow-up. Unfortunately, seven cases died during their acute stage (17.94%) of disease (Figure 8). 

**Figure 9:**
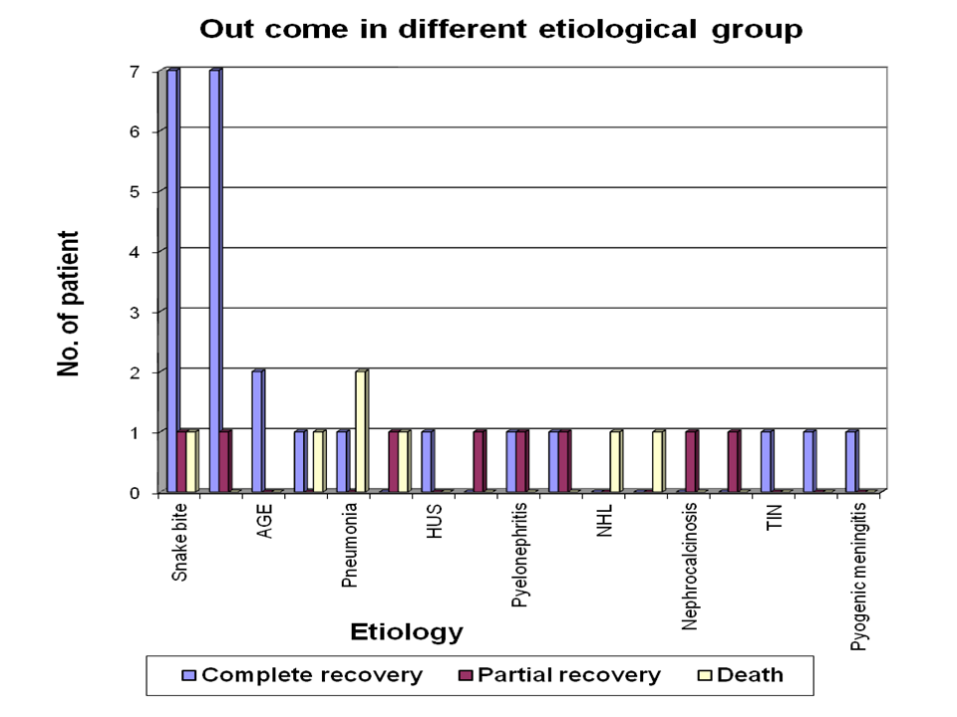
Outcome of complications of acute renal failure in different etiological group

Out of total nine cases in snake envenomation group, seven cases (77.8%) recovered fully, one patient (11.1%) died during acute phase due to DIC and another one child (11.1%) had persistent biochemical abnormality during follow-up. Among the total eight cases of acute renal failure following PSGN, seven patients (87.5%) recovered completely. One child (12.5%) in this group however had persistent biochemical abnormality, though the child was improving until the last follow-up visit. No patient died in this group (Figure 9). 

**Figure 10:**
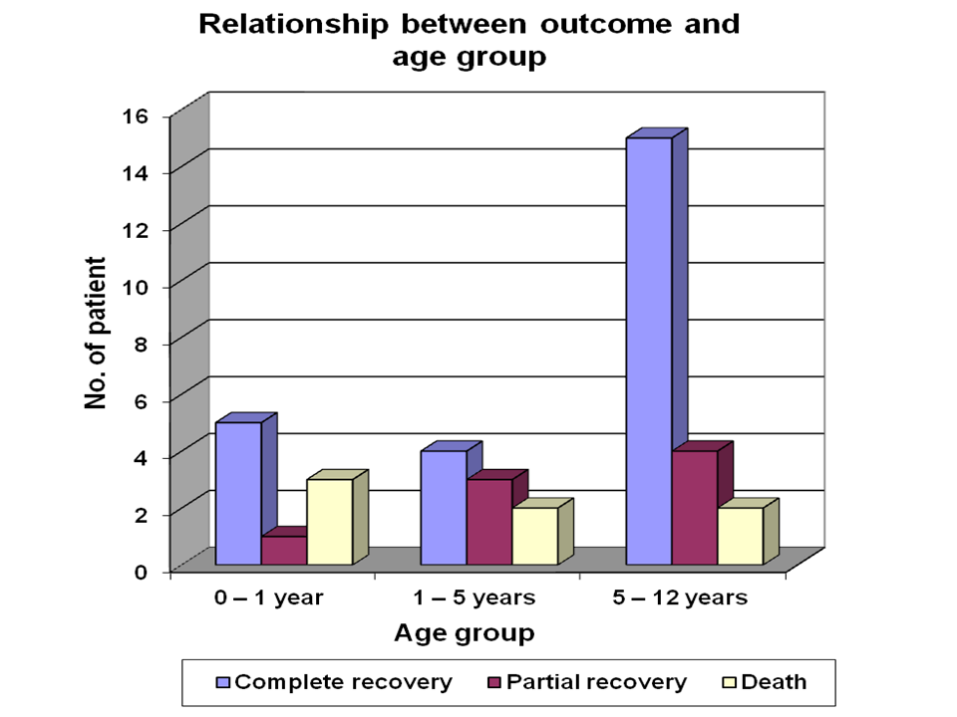
Relationship between outcome and age group in acute renal failure patients

The outcome in the different age group is depicted in Figure 10. Out of total nine patients in zero to one year's age group, five (55.5%) infants recovered completely, three (33.3%) cases died and one case (11.11%) had a partial recovery. Of the nine children in one to five years age group, four cases (44.4%) had the complete recovery, three cases (33.3%) had partial recovery and two cases (22.2%) died. Among the five to 12 years group, 15 cases (71.4%) recovered completely, four cases (19.04%) had the persistent biochemical abnormality and two cases (9.52%) died. 

**Figure 11:**
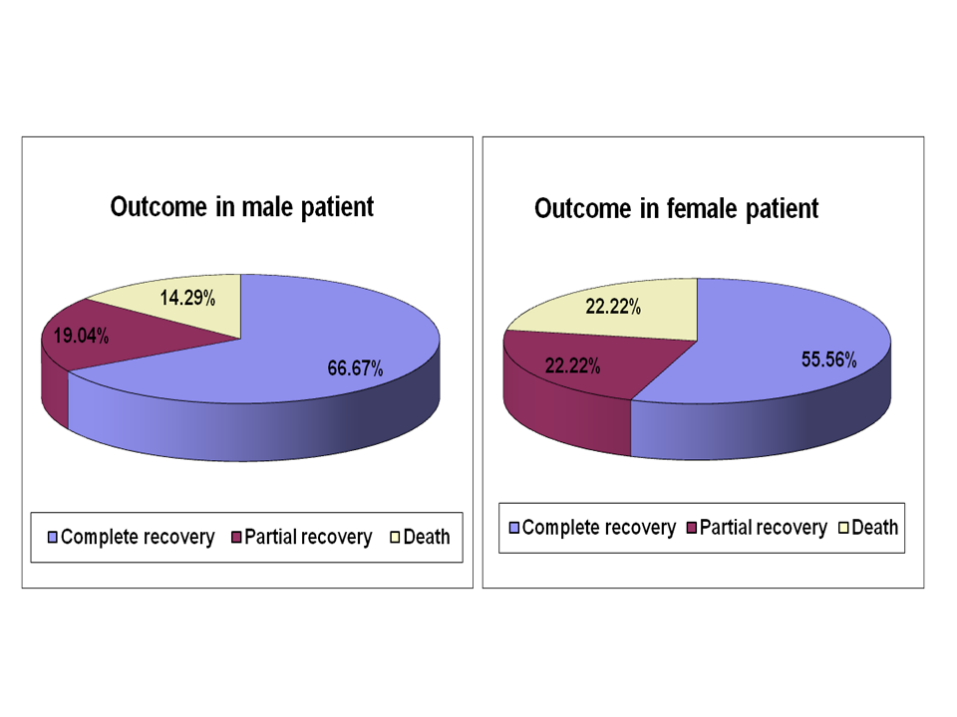
Outcome of acute renal failure in different sex group

The outcome in different sex group is given in Figure 11. Out of total 21 male children, 14 cases (66.67%) recovered completely, four cases (19.04%) had partial recovery and three patients died. Among the 18 female children with ARF, ten cases (55.56%) had a complete recovery, four cases (22.22%) had partial recovery and four cases (22.22%) died. 

**Figure 12:**
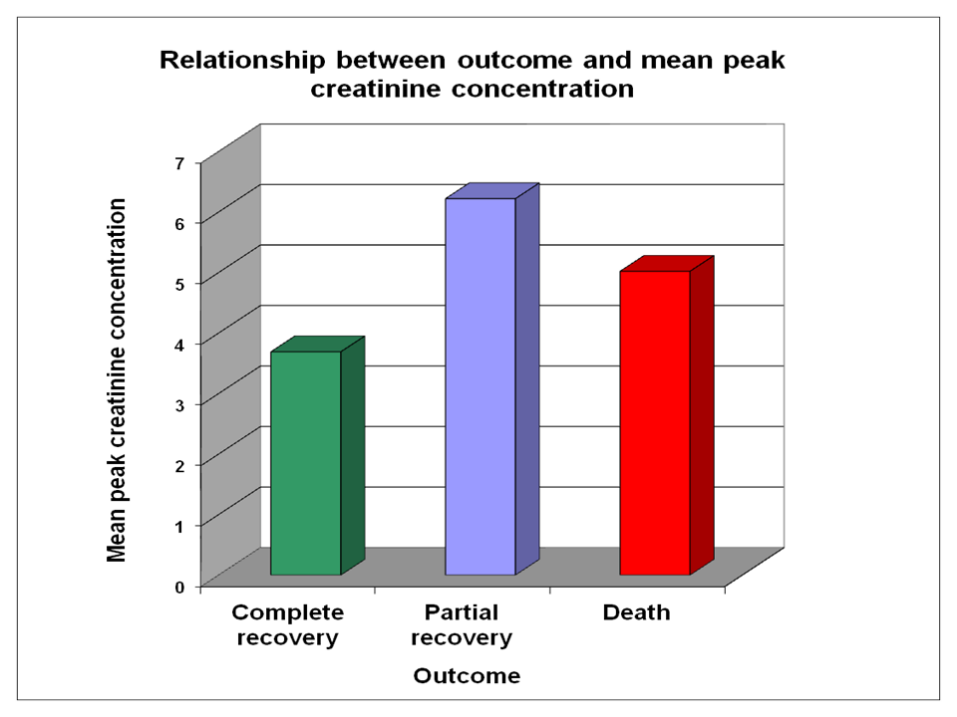
Relationship between outcome and mean creatinine concentration

The relationship between outcome and mean peak creatinine concentration during acute illness is given in Figure 12. In completely recovered group (24 cases), the mean peak creatinine concentration was 3.69 mg/dl. In partially recovered group (eight cases), the mean peak creatinine concentration was 6.22 mg/dL and in patients who died, mean peak creatinine concentration was 5.02 mg/dL. 

Out of 24 cases in the completely recovered group, 14 cases (58.3%) had one or more complications. Among the seven patients, who died during their acute illness, six cases (85.7%) had complications and all the patients (100%) in the partially recovered group had some complications. 

## Discussion

ARF is not uncommon in a pediatric population and has significant morbidity and mortality. The emphasis on presentation remains cardinal, but early detection and appropriate treatment can also provide complete recovery in a large proportion of ARF in children, which is the major goal of ARF therapy. The incidence of ARF in NICUs reported in some previous studies ranges from 6% to 24% of newborns [9]. Although the precise incidence of acute renal failure in children is unknown, studies suggest that the incidence of acute renal failure in hospitalized children may be increasing [10]. In a 22 years study conducted [11], the incidence rate of ARF among children admitted to hospital has dramatically increased from 0.5 – 3.3 cases for 1000 cases before 1995 to 4.6- 9.9 cases per 1000 after 1995. Even though other studies reported an incidence rate less than 1% in 2000 [12], our study showed a higher value of 2.6%, which may be due to the fact that our hospitals are tertiary care hospital and had the facility of renal replacement therapy.

In this study, out of total 39 cases, 21 cases were males and 18 cases were females, and the male-female ratio (M: F) was 1.2:1. This male preponderance was in consistent with previous studies, where always a higher incidence in males was reported [13-17]. ARF in children was most commonly found in five to 12 years age group (53.8%). Infant (zero to one year) and pre-school (one to five years) children contributed 23.08% each separately. Mean age of presentation was 6.14 years, which was in consistent with previously reported studies [14, 16- 17]. The youngest case in our study was a 12-day old newborn male presented with ARF due to PUV. Three children presented at the age of 12 years, two of them had SLE (both female) and the third one, a male child had ARF due to snake envenomation.

Most common cause of ARF in this study was snake envenomation (23.07%) followed by acute post-streptococcal glomerulonephritis (20.5%). Other causes included pneumonia (7.7%), SLE (5.1%), PUV (5.1%), acute gastroenteritis (AGE) (5.1%), hemolytic uremic syndrome HUS (2.6%) etc. In developing countries, volume depletion from diarrhea continues to be the most common cause of ARF [18]. Few previously published studies reported that the most commonly observed cause of ARF was hypovolemia associated mostly with either diarrhea or with HUS [15, 19]. But in the present study, we found only two cases of AGE resulting in 5.1% of ARF among the total 39 cases, which is far lower than the above-mentioned previous reports. This discrepancy may be due to the fact that, all previous studies were reported more than two decade before when the oral rehydration solution (ORS) therapy had not started or was in its nascent stage. As the awareness of ORS therapy from the early stage of diarrhea increased and after our National Diarrhea Control Program (NDCP) launched, there was a drastic decrease in the incidence of diarrhea with severe dehydration.

HUS was one of the common etiologies of ARF in children in many previous studies (12% to 24%), along with acute tubular necrosis and glomerulonephritis [13, 20]. In contrast to their reports, in our study, we found only one case (2.56%) of HUS leading to ARF in our study. This discrepancy could be due to the fact that, HUS is more commonly associated with Escherichia coli in developed countries and Shigella dysenteriae type 1 in developing country. So HUS is more common during the epidemic outbreak of diarrhea due to these organisms. Moreover, a seasonal variation and genetic difference in susceptibility of HUS are also reported in different ethnic groups. Another reason may be that prognosis among HUS is good and most post-diarrheal HUS recovered only by supportive therapy. As diarrheal cases are usually not referred to our hospital frequently and the large proportion of HUS induced mild renal dysfunction and are not recognized in most cases at peripheral level due to lack of familiarity with this etiology, many cases of HUS induced mild ARF are overlooked.

Snake envenomation has contributed a substantial bulk to the etiology of ARF and has been shown in other tropical countries like Asia and Africa [21-23]. In our study, snake bite was the most common etiology accounting for 23.07% of all cases of ARF and was most frequently seen in five-12 years age group (eight out of nine cases). In our country, Hawd region has the highest incidence of snake bite cases [24] and children as the common victims with an incidence rate of 7% to 22% [25-27]. Though ARF is a well-known complication of snakebite among children, snake envenomation has not frequently been mentioned as the major cause of ARF in children. As this study is done in a region where the incidence of snake bite is very high and most of the peripheral centers have the scarcity of snake anti-venom serum, patients often do not get adequate treatment. On the other hand, like most developing countries, a large proportion of rural cases first consult a traditional practitioner before visiting a medical center, which further enhances the chances of developing ARF due to delay in treatment. Lastly, most of the snake bite cases with ARF complications from peripheral centers are referred to our institution for dialysis and better management. Probably, all these factors are responsible for this large proportion case of ARF due to snake envenomation in our study.

The second most common etiological factor responsible for ARF in this study was acute post-streptococcal glomerulonephritis (APSGN), which contributed 20.5% and was in consistent with previous reports [15]. In our study, among the APSGN group, male were slightly predominant than female cases and most (75%) cases were in the five to 12 years age group, which is similar to a study conducted by Vachvanichsanong, et al. (71% in five to 13 year age group) [11], 87.5% cases recovered completely and only one patient had persistent renal dysfunction without any mortality.

Overall outcome in our study has been recorded in three categories as completely recovered, partially recovered (with some persistent clinical or biochemical abnormality) or the patient died during the acute stage of the disease. The overall mortality in our study (17.9%) was lower than many previously published reports [17, 28]. Although mortality depends mainly on the etiology, other factors such as study population, the age of patient, medical and dialysis facility also influenced the mortality rate. A large proportion of cases were due to APSGN (20.5%), which showed 100% survival rate, further contributed to lower mortality in our study. Contrarily, a short follow-up period of cases might have resulted in a low mortality recording, because partially recovered cases may ultimately develop the end-stage renal disease in long-term follow-up and died if transplantation not done.

Mortality in infants (zero to one year age group) was higher (33.3%) than the other two groups (22.2% in partially recovered cases and 9.5% in five to 12 years age group), which was consistent with other studies [29]. Mortality in the female child was slightly higher than the male child (14.3%). Similarly, mortality was higher in patients who had higher mean peak creatinine concentrations (6.22 mg/dl in the partially recovered group and 5.02 mg/dl in death group) and in children who developed complications during their hospital stay. Mortality was very high in children who had complications like DIC (100%), arrhythmia (100%), seizure (33.3%) and pneumonia (33.3%).

## Conclusions

The precise incidence of acute renal failure in the pediatric patient is difficult to define, as it largely depends on the referral patterns, proximity to pediatric renal unit and expertise within the unit. It also depends on the population studied and the geographic location of the study. A large number of pediatric patients in our country, mainly from rural areas are not adequately managed because of poverty and lack of facilities for dialysis. Many causes of ARF in our environment are preventable and it should be possible to reduce mortality and morbidity due to ARF through purposive preventive measure and availability of the better medical facility.

Our study was comparatively of short duration with the small number of cases. The follow-up was done for a very short period of time. Therefore, it was very difficult to conclude an incidence, long-term mortality, morbidity and other variables from the present study. So, it is essential to conduct a large multi-centric long-term follow-up assessment of children’s clinical condition and renal function study.
